# How golden age Arab-Islamic scholars revolutionized sleep physiology and dream analysis

**DOI:** 10.1016/j.jtumed.2025.07.013

**Published:** 2025-09-05

**Authors:** Ahmed S. BaHammam

**Affiliations:** a Department of Medicine, College of Medicine, University Sleep Disorders Center, King Saud University, Riyadh 11324, KSA; b The Strategic Technologies Program of the National Plan for Sciences and Technology and Innovation in the KSA, Riyadh, KSA; c King Saud University-Medical City (KSU-MC), Riyadh, KSA

**Keywords:** تاريخ طب النوم, الطب الإسلامي, العلوم في العصور الوسطى, ابن سينا, الرازي, الأحلام, الحضارة العربية, Al-Rāzī, Dreams, Ibn Sīnā, Islamic medicine, Medieval science, Arab civilizationِ, Sleep medicine history

## Abstract

Medieval Arab-Islamic scholars (7th–13th centuries CE) made pivotal but underacknowledged contributions to sleep science, blending empirical observation with theological insight. This review examines primary texts, especially al-Rāzī's *al-Ḥāwī fī al-Ṭibb* and Ibn Sīnā's *Canon of Medicine*, alongside secondary literature to illuminate their advanced understanding of sleep physiology, disorders, and dreams. Al-Rāzī pioneered early clinical methodologies, using comparative groups to evaluate treatments for sleep disorders. He offered detailed observations of *al-kābūs* (sleep paralysis), distinguishing between gastric and brain-centered types, and addressed conditions such as insomnia and nocturnal enuresis. His sleep hygiene recommendations, emphasizing diet, routine, and emotional balance, align strikingly with modern best practices. Ibn Sīnā's pneumatic theory of the *ruh nafsani* (psychic spirit) provided a mechanistic explanation for sleep-wake transitions and proposed three sleep stages based on pulse changes. He also provided an early and clinically relevant description of symptoms consistent with obstructive sleep apnea, recommending positional therapy. His analyses of sleep posture, digestion, and pharmacology demonstrate a systemic approach that is still relevant today. Ibn al-Nafīs expanded sleep theory by arguing that internal faculties like imagination remain active during sleep, prefiguring modern understandings of selective neural activation. Ibn al-Jazzār described hypersomnia (*subāt*) and epilepsy-related collapse, hinting at early notions of narcolepsy. Islamic dream theory, grounded in the Qur'an and Prophetic traditions, recognizes dreams as both physiological phenomena and spiritual messages. Medieval Arab civilization scholars developed and refined classification systems that distinguished divine visions from psychologically and physically induced dreams, an approach that bridged theology, philosophy, and proto-psychology. Together, these contributions demonstrate that medieval Islamic scholars laid key foundations for sleep medicine, challenging Eurocentric histories and affirming the enduring value of diverse intellectual traditions.

## الملخص

قدّم علماء العرب المسلمين في العصور الوسطى (من القرن السابع إلى الثالث عشر الميلادي) مساهماتٍ محوريةً في علم النوم، لم تُقدّر حق قدرها، مزجوا فيها بين الملاحظة التجريبية والفهم الفقهي. تتناول هذه المراجعة النصوص الأساسية، وخاصةً كتاب "الحاوي في الطب" للرازي وكتاب "القانون في الطب" لابن سينا، إلى جانب المراجع الثانوية، لإلقاء الضوء على فهمهم المتقدم لفسيولوجيا النوم واضطراباته والأحلام. كان الرازي رائدا في المنهجيات السريرية المبكرة، مستخدما مجموعات مقارنة لتقييم علاجات اضطرابات النوم. قدّم ملاحظات مُفصّلة عن "شلل النوم"، مُميّزا بين الأنواع المُتمركزة في المعدة والدماغ، وتناول حالات مثل الأرق وسلس البول الليلي. تتوافق توصياته المتعلقة بالممارسات الصحية في النوم، والتي تُركّز على النظام الغذائي والروتين والتوازن العاطفي، بشكلٍ لافت مع أفضل الممارسات الحديثة. قدمت نظرية ابن سينا النفسية (الروح النفسية) تفسيراً ميكانيكياً لانتقالات النوم واليقظة، واقترح ثلاث مراحل للنوم بناءً على تغيرات النبض. كما قدم وصفاً مبكراً وذا صلة سريرية لأعراض انقطاع النفس الانسدادي النومي، موصيا بالعلاج الوضعي. تُظهر تحليلاته لوضعية النوم، والهضم، وعلم الأدوية نهجاً منهجياً لا يزال صالحاً حتى اليوم. كذلك، وسّع ابن النفيس نظرية النوم مجادلا بأن القدرات الداخلية، مثل الخيال، تظل نشطة أثناء النوم، مما مهّد الطريق لفهم حديث للتنشيط العصبي الانتقائي. وصف ابن الجزار فرط النوم (السبات) والانهيار المرتبط بالصرع، ملمحا إلى المفاهيم المبكرة لمرض النوم القهري (الانتيابي). تُقر نظرية الأحلام الإسلامية، المستندة إلى القرآن الكريم والأحاديث النبوية الشريفة، بالأحلام كظواهر فسيولوجية ورسائل روحية. طوّر علماء الحضارة العربية في العصور الوسطى أنظمة تصنيف دقيقة ميّزوا من خلالها بين الرؤى الإلهية والأحلام الناتجة عن أسباب نفسية أو جسدية، وهو نهج جمع بين علم اللاهوت، والفلسفة، والبدايات الأولى لعلم النفس.. تُبيّن هذه المساهمات مجتمعةً أن علماء المسلمين في العصور الوسطى وضعوا أسسا أساسية لطب النوم، مُتحدّين بذلك التاريخ ذا النزعة الأوروبية، ومؤكدين على القيمة الراسخة للتقاليد الفكرية المتنوعة.

## Introduction

The scientific study of sleep has evolved noticeably over the past century, transforming from philosophical speculation to rigorous empirical investigation. However, conventional historical accounts often present an incomplete narrative that jumps directly from ancient Greek medicine to European Renaissance scholarship, effectively erasing the substantial contributions of medieval Arab-Islamic civilization.^1^^,^^2^ This historical gap represents more than mere academic oversight; it perpetuates a Eurocentric perspective that fails to acknowledge the sophisticated understanding of sleep and dreams developed by scholars working within the Islamic cultural sphere between the 7th and the 13th centuries CE.

The Arabic/Islamic perspective on sleep derives from fundamental theological principles. The Quran describes sleep as one of God's signs: “*And among His signs is your sleep by night and day and your seeking of His bounty. Indeed, in that are signs for a people who listen*” (Quran 30:23). This spiritual framework encouraged empirical investigation rather than discouraging it, leading numerous Muslim scholars to examine sleep as both a divine phenomenon worthy of contemplation and a natural process amenable to scientific analysis.^3^, ^4^, ^5^

During the medieval period, Arab civilization experienced unprecedented intellectual expansion (7th–13th century), extending across three continents from China to Spain 6. This geographical scope facilitated the synthesis of diverse medical traditions, including Greek, Persian, Indian, and indigenous Arabian healing practices. Arab-Islamic physicians not only preserved and translated ancient texts but substantially advanced medical knowledge through original research, systematic observation, and innovative theoretical frameworks.^7^

The Mongol invasion and sack of Baghdad in 1258 CE marked one of the most devastating episodes in the intellectual history of the Islamic world. Contemporary accounts describe the mass destruction of libraries and the loss of countless manuscripts, with one report claiming the Tigris River ran black from the ink of discarded books.^8^ Historians widely view this event as a turning point that disrupted centuries of scholarly development and led to a long-term decline in scientific production in the region.^9^^,^^10^ This catastrophic rupture helps explain why early Arab contributions to fields like sleep medicine are underrepresented in modern historical accounts. Nevertheless, surviving texts reveal a sophisticated understanding of sleep physiology, sleep disorders, and therapeutic interventions that anticipated many developments in contemporary sleep medicine.

This review provides a comprehensive analysis of medieval Arab-Islamic contributions to sleep science, focusing particularly on the works of prominent physicians such as Al-Rāzī (Rhazes) and Ibn Sīnā (Avicenna). By examining their theoretical frameworks, clinical observations, and therapeutic recommendations, we seek to demonstrate that these scholars established fundamental principles that continue to inform modern sleep medicine. Additionally, this analysis explores how Arab-civilization scholars approached dreams and their interpretation, revealing complex theoretical systems that integrated empirical observation with spiritual understanding.

## Overview of review structure

This review is organized chronologically and thematically to trace the evolution of sleep medicine in medieval Arab-Islamic civilization.

• **Historical Foundations** (Section 3): Greek antecedents and methodological approach
• **Early Pioneers** (Sections 4-5): Al-Rāzī's clinical contributions (865–925 CE)
• **Theoretical Advances** (Section 6): Ibn Sīnā's pneumatic theory and systematic approach (980–1037 CE)
• **Later Developments** (Section 7): Ibn al-Nafīs, Ibn al-Jazzār, and other scholars (10th-13th centuries)
• **Institutional Context** (Section 8): Knowledge transmission networks
• **Dream Theory Integration** (Section 9): Theological and psychological frameworks
• **Clinical Applications** (Section 10): Therapeutic approaches and modern relevance

**Figure 1:**
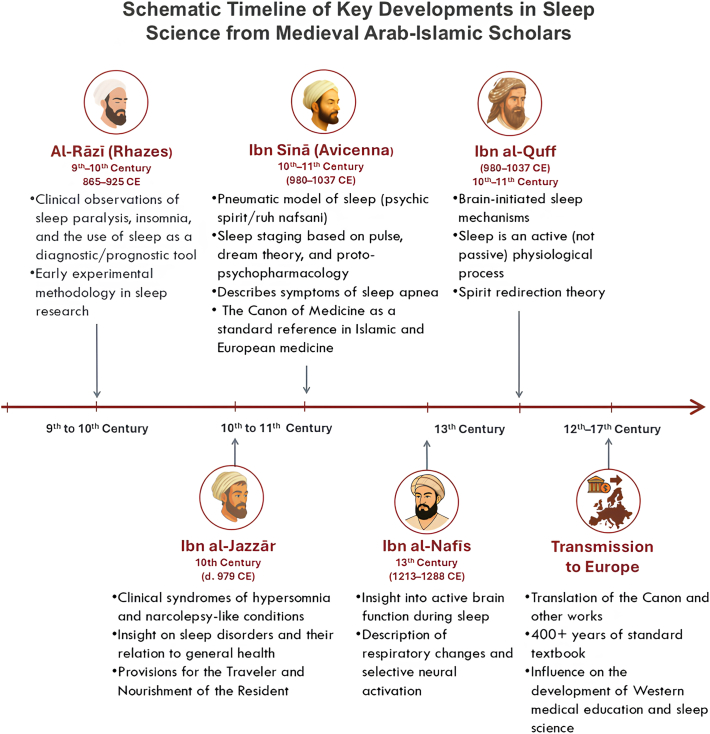
Schematic Timeline of Key Developments in Sleep Science from Medieval Arab-Islamic Scholars (8th–13th Centuries CE). (No permission is needed).

The sophisticated understanding of sleep medicine developed by medieval Arab-Islamic civilization scholars resulted from the contributions of several key figures whose work spanned multiple centuries and geographical regions (Figure 1). These scholars, working within major medical centers from Baghdad to Damascus to Kairouan and Persia, established foundational principles that influenced both Islamic and European medical practice for centuries.

## Historical foundations: Greek antecedents

Before examining Arab-Islamic innovations, it is essential to understand the Greco-Roman foundations upon which these scholars built their work. Aristotle (384–322 BCE) proposed that sleep occurs when the master sense organ located in the heart becomes incapacitated, thereby ceasing all sense perception.^2^ Despite this cessation of external awareness, Aristotle recognized that the body remained metabolically active during sleep, particularly in completing the transformation of food into pure blood. He observed that innate heat turned inward and moved to the lower regions of the body to accomplish this digestive process, which explained why the master sense organ ceased its activity since innate heat was the driving force for all bodily activities.^2^

Galen (129–216 CE) challenged Aristotle's cardio-centric model through anatomical investigations that demonstrated the brain's role in sensation, movement, and cognitive functions, including memory and imagination.^2^ Galen theorized that sleep served the brain's need to restore its perceptual powers. According to his model, sleep helps the brain recover by temporarily withdrawing the perceptual faculty, leading to brain and nerve moistening while relaxing sensory perception rather than ceasing entirely. Galen noted that sensation relaxes progressively during sleep, with deeper sleep corresponding with greater sensory reduction. Galen also noted that innate heat concentrated internally toward the heart and stomach to facilitate digestion, although he attributed the digestive process to the nutritive part of the soul associated with the liver.^2^

These Greco-Roman theories provided the conceptual starting point for Arab-Islamic physicians, who both preserved this knowledge and substantially extended it through empirical observation and theoretical innovation.

## Methodological approach

### Database search strategy

Our systematic literature search was conducted across six major academic databases between January 2023 and April 2025, yielding a total of 2847 initial results. Searches were conducted across several major academic databases including PubMed/MEDLINE, Google Scholar, JSTOR, Cambridge Core, Brill Online, and the Qatar National Library. These searches utilized combinations of keywords including “Islamic medicine,” “medieval sleep,” “Ibn Sina,” “Avicenna,” “al-Razi,” “Rhazes,” and “Arab medicine” in both English and Arabic. Google Scholar searches employed both English and Arabic terms to capture interdisciplinary academic sources. Relevant sources were identified through a systematic screening of titles, abstracts, and full texts across all databases, resulting in a curated selection of primary and secondary literature that focuses on medieval Arab-Islamic contributions to sleep medicine and related fields.

Our search strategy employed systematic Boolean combinations in both English and Arabic to ensure comprehensive coverage of the relevant literature. English search terms included: (“Islamic medicine” OR “Arab medicine” OR “medieval medicine”) AND (“sleep” OR “dreams” OR “insomnia” OR “sleep disorders”), (“Ibn Sina” OR “Avicenna”) AND (“sleep” OR “pneumatic theory” OR “Canon of Medicine”), (“al-Razi” OR “Rhazes”) AND (“sleep paralysis” OR “nightmare” OR “sleep hygiene”), and (“medieval Islamic” OR “Arab Islamic”) AND (“sleep physiology” OR “dream interpretation” OR “sleep apnea”). Arabic search terms included systematic combinations of "الطب الإسلامي" (Islamic medicine), "النوم" (sleep), "الأحلام" (dreams), "ابن سينا" (Ibn Sina), "الرازي" (al-Razi), and "الطب العربي" (Arab medicine). Database-specific filters were applied to limit the results to peer-reviewed articles, academic books, and scholarly dissertations published between 1950 and 2025, ensuring both historical depth and contemporary relevance.

Primary sources were systematically selected based on specific inclusion criteria: temporal coverage (650–1300 CE), geographical scope (major Islamic intellectual centers including Baghdad, Cairo, Damascus, Cordoba, and Kairouan), content relevance (explicit discussions of sleep physiology, disorders, or dream interpretation), historical significance (influence on subsequent medical practice), and manuscript availability (accessible Arabic originals or reliable critical editions). Exclusion criteria eliminated sources lacking peer review, popular publications without scholarly rigor, secondary sources without primary text citations, works focused solely on non-sleep medical topics, and sources with inadequate historical documentation. From the initial 2847 database results, 1358 were excluded during title screening, 743 during abstract review, and 567 during full-text assessment, resulting in 179 sources meeting all inclusion criteria.

### Thematic coding

Categorized extracted data into sleep physiology theories, sleep disorder classifications, diagnostic approaches, therapeutic interventions, and dream interpretation frameworks. Contemporary validation was achieved through a systematic literature review of modern sleep medicine (2000–2024), with an emphasis on physiological mechanisms, diagnostic criteria, and therapeutic approaches. Language protocols included direct consultation of original Arabic manuscripts, cross-referencing multiple manuscript traditions, verification through classical Arabic dictionaries, and cross-validation of translations against authoritative sources. For more details about the methods used, refer to the Supplementary Methodology.

## Arab civilization contributions to sleep medicine: an overview

This review employs civilizational terminology rather than ethnic classification, following established scholarly conventions in the history of science. The term ‘Arab-Islamic civilization’ refers to the cultural, linguistic, and institutional framework within which these scholars operated, regardless of their ethnic origins. This approach aligns with standard academic practice in civilizational studies, such as ‘Greco-Roman civilization’ or ‘Chinese civilization,’ which encompass diverse ethnic contributions within unified cultural matrices.^11^ The focus remains on the intellectual and institutional context that enabled these scientific advances rather than individual ethnic identities.

### Arabic as scientific lingua franca

From the late Umayyad period (661–750 CE) and even more so under the Abbasids (750–1258 CE), Arabic functioned as the institutional language of administrative governance, medical education, scholarly discourse, and knowledge transmission through hospital-based training programs.^12^ This linguistic unity created a shared intellectual framework that transcended ethnic boundaries, enabling scholars from diverse backgrounds to contribute to a unified tradition of medical practice and research within Arabic-speaking institutional networks.

**Figure 2:**
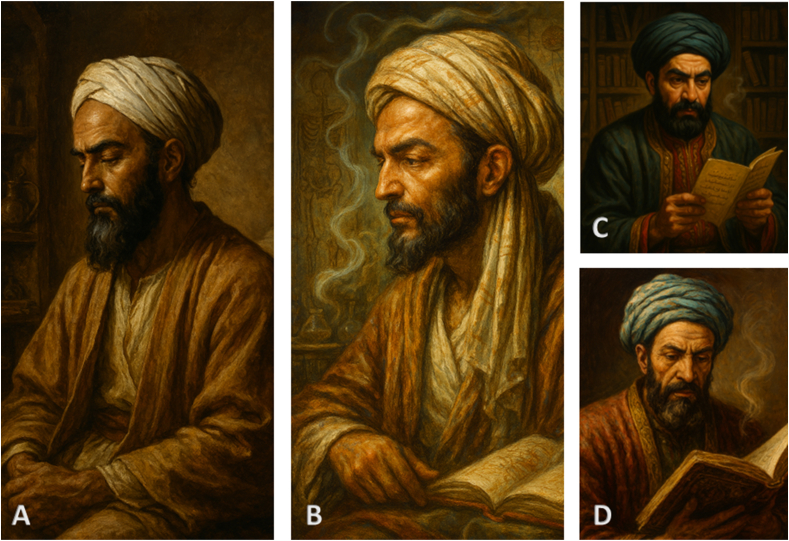
Key Contributors to Medieval Arab-Islamic Sleep Medicine. **(A)** Al-Rāzī (865–925 CE) served as chief physician of Baghdad's Bimaristan and pioneered clinical sleep medicine through experimental methodology and systematic descriptions of sleep paralysis (al-kābūs). **(B)** Ibn Sīnā (980–1037 CE), court physician in Bukhara and vizier, known as the “Prince of Physicians,” developed the revolutionary pneumatic theory of sleep and created the first systematic sleep stage classification in his influential Canon of Medicine. **(C)** Ibn al-Nafīs (1213–1288 CE) practiced in Damascus and advanced understanding of sleep physiology by demonstrating that internal brain faculties remain active during sleep while external senses are suspended. **(D)** Ibn al-Jazzār (died 979 CE) led the Kairouan medical school and provided early clinical descriptions of hypersomnia (subāt) and sleep-related disorders in his medical compendium. (No permission is needed).

The scope of Arab civilization's contributions to sleep and sleep medicine extends far beyond the mere preservation of ancient knowledge. These scholars established original research programs, developed innovative theoretical models, and created comprehensive clinical approaches to sleep disorders that remained influential well into the early modern period. Figure 2 provides a comprehensive overview of the key medieval Arab-Islamic physicians and their specific contributions to sleep science, demonstrating the breadth and sophistication of their innovations across multiple centuries.

## Al-Rāzī (865–925 CE): clinical foundations and contributions to sleep medicine

**Figure 3:**
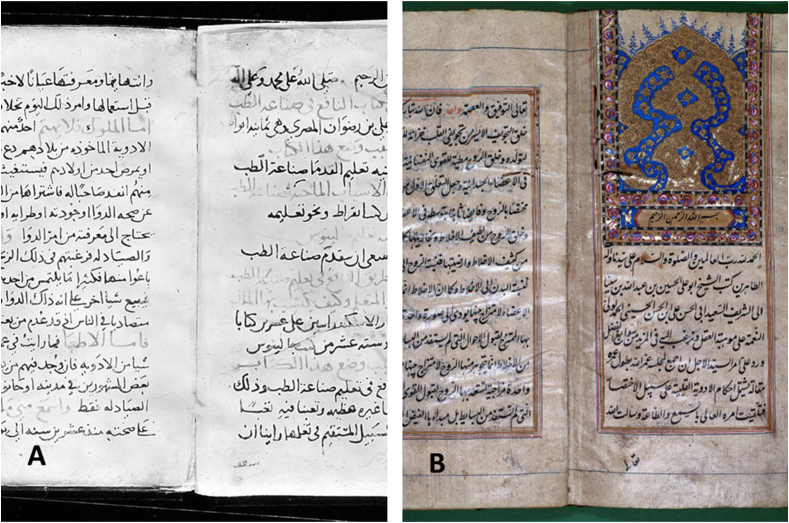
Primary source manuscripts from key medieval Arab-Islamic medical texts. (A) Manuscript folio from Al-Rāzī's “Kitāb al-Ḥāwī fī al-Ṭibb" (The Comprehensive Book), demonstrating the Arabic medical textual tradition and scholarly documentation methods used in medieval Islamic medicine. (B) Illuminated opening page of Ibn Sīnā's “al-Qānūn fī al-Ṭibb" (Canon of Medicine), showcasing the sophisticated artistic and scholarly presentation of medieval Islamic medical manuscripts. Both manuscripts exemplify the rich intellectual tradition of Arab-Islamic medical scholarship that established foundational contributions to sleep science and general medicine. Source: Wellcome Collection. Licensed under Creative Commons Attribution 4.0 International License (CC BY 4.0).

Abu Bakr Muhammad ibn Zakariya al-Rāzī (865–925 CE), known in the Latin West as Rhazes, stands among the most influential physicians in Islamic medical history. His monumental work “*Kitāb al-Ḥāwī fī al-Ṭibb"* (often referred to as “The Comprehensive Book” or “The Continens” in English) represents one of the largest medical encyclopedias ever written, encompassing detailed discussions of sleep disorders alongside systematic clinical observations (Figure 3A).^13^

### Sleep as a diagnostic and prognostic indicator

#### Clinical methodology: sleep as a diagnostic and prognostic indicator

Al-Rāzī pioneered the clinical use of sleep patterns as diagnostic and prognostic tools. In his “Royal Book of Medicine” (*al-Tibb al-Muluki*), he emphasized that restoration of normal sleep following illness represents a reliable indicator of recovery: “When a patient sleeps natural sleep after severe insomnia, this indicates the breaking of fever and the body's return to health”.^14^

This clinical insight demonstrates an early understanding of the potential relationship between sleep and immune function, anticipating modern research demonstrating sleep's crucial role in supporting immune responses and accelerating healing processes.^15^^,^^16^ While contemporary medicine relies on advanced polysomnographic techniques, al-Rāzī's emphasis on careful clinical observation of sleep changes provided an early example of evidence-based diagnostic practice.

### Experimental methodology in sleep research

Al-Rāzī employed experimental methodology in studying sleep disorders and their treatments. His approach to investigating meningitis illustrates this systematic approach: “When heaviness and pain in the head and neck persist for three, four, five days or more, and the patient avoids looking at light, with copious tearing, extensive yawning and stretching, and severe insomnia causing extreme fatigue, the patient will develop meningitis … When you observe these symptoms, immediately perform bloodletting. I have saved one group of patients with this treatment, while I deliberately neglected bloodletting in another group. Through this approach, I sought to reach a conclusion. Thus, all of the latter group developed meningitis”.^13^

This methodological approach represents remarkable sophistication in experimental design, including using comparison groups to evaluate treatment efficacy, a principle that would not become standard in Western medicine until centuries later.

### Sleep paralysis and the “nightmare” phenomenon

Medieval understanding of sleep paralysis, known in Arabic medical literature as “al-kabous” (the nightmare), demonstrates the advanced clinical observation skills of Arab civilization physicians. While European sources often attribute the first medical description of sleep paralysis to Dutch physician Isbrand van Diemerbroeck (1609–1674), al-Rāzī provided detailed clinical descriptions centuries earlier in his “Comprehensive Book”.^17^

Al-Rāzī wrote: “When the nightmare occurs, the person feels something heavy pressing upon them and finds themselves unable to cry out”.^18^ This description accurately captures the core features of sleep paralysis as understood in contemporary sleep medicine. Al-Rāzī further distinguished between two types of sleep paralysis based on underlying pathophysiology: gastric dysfunction caused by vapors rising from the stomach to the brain, particularly following consumption of heavy foods before sleep, and brain-centered disturbances characterized by more severe and persistent symptoms related to brain temperament imbalances.

Al-Rāzī’s clinical approach to sleep paralysis (*al-kābūs*) was notably advanced for his era, as he distinguished between two principal types based on their underlying causes: “gastric” and “brain centered”.^1^^,^^18^^,^^19^

The “gastric” type of sleep paralysis was attributed to disturbances originating in the digestive system. He theorized that consuming heavy or indigestible foods, especially in the evening, could result in vapors or noxious substances rising from the stomach to the brain during sleep. These vapors, he believed, interfered with normal brain function and triggered episodes of paralysis upon falling asleep or awakening. To address this form, al-Rāzī recommended a regimen focused on dietary moderation, specifically, avoiding large or rich meals before bedtime, and the use of herbal remedies known to soothe the stomach and aid digestion. This preventative strategy reflects his understanding of the close relationship between gastrointestinal health and sleep quality.^1^^,^^19^ On the other hand, the “brain-centered” type was considered by al-Rāzī to arise from an intrinsic disorder of the brain itself, often associated with what he described as a “cold temperament” in cerebral tissues. This form was thought to be more severe and persistent than the gastric type. For these cases, al-Rāzī prescribed interventions aimed at “warming” the brain, such as the application of warming agents, and in some situations, selective bloodletting to rebalance bodily humors. He also recommended specific medications intended to correct the brain's temperament, reflecting the humoral theory prevalent in medieval medicine.^18^^,^^19^

Although the proposals by al-Rāzī are not directly supported by recent literature, this dual classification and tailored therapeutic approach demonstrate al-Rāzī’s sophisticated clinical analysis to understand this phenomenon. By differentiating between peripheral (gastric) and central (brain) origins of sleep paralysis, and by prescribing targeted interventions for each, al-Rāzī anticipated core principles of modern sleep medicine, which emphasize identifying and treating the root cause of sleep disorders.^1^ Table 1 illustrates how medieval Arab-Islamic classifications of sleep disorders correspond remarkably well with modern diagnostic categories, revealing sophisticated clinical observation skills that enabled accurate symptom recognition and targeted therapeutic approaches.

**Table 1:** Medieval Arab-Islamic Classification of Sleep Disorders and Modern Equivalents.

Medieval Term/Description	Source/Physician	Modern Equivalent	Pathophysiological Insight	Recommended Therapy
Al-kābūs (sleep paralysis, nightmare)	Al-Rāzī, Akhawayni	Sleep paralysis	Gastric vs. brain-centered causes	Dietary regulation, herbal remedies, and bloodletting
Subāt (torpor, hypersomnia)	Ibn al-Jazzār	Hypersomnia, narcolepsy	Excess cold/phlegm in the brain	Humoral balancing, lifestyle modification
Insomnia with fever/meningitis	Al-Rāzī	Insomnia secondary to illness	Neurological/psychiatric link	Disease-specific therapy, sleep hygiene
Nocturnal enuresis	Al-Rāzī	Nocturnal enuresis	Bladder sphincter relaxation	Fluid restriction, herbal remedies, and urethral medications
Symptoms of obstructive sleep apnea (back sleeping, snoring, choking)	Ibn Sīnā	Obstructive sleep apnea	Tongue relaxation, muscle weakness	Side sleeping, positional therapy

Contemporary physician Abu Bakr Rabi ibn Ahmad al-Akhawayni al-Bukhari (died 983 CE) challenged supernatural explanations of sleep paralysis in his *Hidayat al-Mutaʽallemin fi al-Ṭibb* (*A Guide to Medical Learners*).^20^ He provided physiological explanations, describing sleep paralysis as “a precursor to epilepsy caused by vapors rising from the stomach to the brain,” particularly affecting individuals with cold brain temperament where cold blood flows in the brain and its vessels.^21^ While not entirely accurate by modern standards, this represents an early attempt to provide naturalistic rather than supernatural explanations for sleep phenomena.

### Nocturnal enuresis

Al-Rāzī also documented nocturnal enuresis in *al-Ḥāwī fī al-Ṭibb* (The Comprehensive Book), explaining that it can occur during deep sleep *(*i.e., *prolonged or very heavy sleep as described by al-Rāzī, not the specific non-rapid eye movement [NREM] stage 3 of modern sleep science)* due to relaxation or weakness of the bladder and anal sphincter muscles. He observed that exposure to cold, such as sitting on cold stones or standing in cold water, may result in involuntary urination and defecation, attributing this to the effect on the pelvic muscles. To prevent this, al-Rāzī advised limiting fluid intake in the evening and avoiding excessively deep or prolonged sleep, especially in susceptible individuals. For severe or persistent cases, he recommended the use of medicinal preparations administered directly into the bladder via the urethra.^1^^,^^19^^,^^22^

### Insomnia and associated disorders

Al-Rāzī's analysis of insomnia revealed an understanding of its relationship with various neurological and psychiatric conditions. He documented the connection between insomnia and meningitis, noting: “If head heaviness exceeds pain and insomnia is absent with sleep present, fever will subside, but the pulse will be tremendous though infrequent, and the patient will develop stupor”.^13^ This demonstrates an appreciation for sleep disturbances' diagnostic and prognostic value in neurological conditions.

In his “Royal Book of Medicine,” al-Rāzī addressed insomnia accompanying various diseases, recognizing the close relationship between sleep and healing. He described sleep disturbances in cardiac conditions and liver diseases, recommending specific dietary interventions to improve sleep quality.^14^ This integrated approach, combining sleep optimization with organ-specific treatments, anticipates a modern understanding of bidirectional relationships between sleep disorders and systemic diseases.^23^^,^^24^

### Dietary influences on sleep

Al-Rāzī devoted considerable attention to the impact of food on sleep quality, categorizing foods as either sleep-promoting (lettuce, squash, violet preparations) or sleep-disrupting (hot and spicy foods). In The Comprehensive Book, he recommended: “Those with disturbed sleep and frequent insomnia should avoid hot and spicy foods and consume moisture-balancing foods like lettuce and squash, bathe in lukewarm water before sleep, take light walks, and avoid troubling thoughts at bedtime. If these measures prove insufficient, sleep-inducing medications such as poppy syrup or violet oil applied to the temples may be used”.^14^^,^^19^

Table 2 summarizes the comprehensive sleep hygiene and dietary recommendations developed by medieval Arab-Islamic physicians, many of which align remarkably well with contemporary evidence-based sleep medicine practices.

**Table 2:** Medieval Sleep Hygiene and Dietary Recommendations.

Recommendation	Physician	Rationale/Mechanism	Modern Evidence/Correlation
Avoid heavy meals before sleep	Al-Rāzī, Ibn Sīnā	Prevent gastric sleep paralysis	Supported by sleep quality research
Sleep after digestion, not on full/empty stomach	Ibn Sīnā	Optimize sleep quality and digestion	Modern sleep hygiene guidelines
Use of lettuce, squash, violet	Al-Rāzī	Sleep-promoting foods	Lactucin in lettuce, sleep studies
Lukewarm bath, light walk	Al-Rāzī	Relaxation, aid digestion	Endorsed in behavioral sleep therapy
Avoid hot/spicy foods	Al-Rāzī	Prevent sleep disruption	Correlated with sleep disturbances

This systematic approach to sleep hygiene through dietary modification anticipates contemporary research demonstrating specific foods' effects on sleep quality and duration.^25^ Al-Rāzī's recommendations regarding lettuce align with modern research identifying sleep-promoting compounds such as lactucin in lettuce.^26^

## Ibn Sīnā (980–1037 CE): theoretical revolution and sleep medicine contributions

Building on al-Rāzī's clinical observations, Ibn Sīnā developed comprehensive theoretical frameworks that transformed the medieval understanding of sleep physiology and established systematic approaches to sleep medicine that remained influential for centuries. Abu Ali al-Husayn ibn Abd Allah ibn Sīnā (980–1037 CE), known in the Latin West as Avicenna, developed the most sophisticated and influential theory of sleep in medieval medicine. His Canon of Medicine (al-Qānūn fī al-Ṭibb) remained a standard medical textbook in both Islamic and European universities for centuries (Figure 3B), with the first Latin edition appearing in 1472 and subsequent editions continuing until 1608.^27^

### The pneumatic model of sleep

Ibn Sīnā's revolutionary contribution to sleep science lies in his pneumatic (hawa'i) theory, which centered on the role of the psychic spirit (ruh nafsani) in explaining sleep mechanisms. Unlike previous Greek theories that focused primarily on heat redistribution or brain moisture, Ibn Sīnā's model provided a comprehensive mechanistic explanation for sleep-wake transitions (Fancy, 2023b).

According to Ibn Sīnā's theory, sleep occurs due to “desiccation and dissolution of the psychic spirit resulting from its continuous movement during wakefulness.” The psychic spirit, which Ibn Sīnā considered the primary instrument through which the soul operates, becomes depleted through daytime activities and requires restoration through sleep. This restoration occurs when the spirit withdraws from sensory and motor instruments to its source (the brain), allowing for renewal while maintaining essential life functions such as respiration.

Ibn Sīnā defined sleep as “the return of the psychic spirit from the instruments of sensation and movement to the source, whereby these instruments cease their actions due to this withdrawal, except for those necessary for survival, such as respiratory instruments”.^2^^,^^28^

This theoretical framework enabled Ibn Sīnā to explain various sleep phenomena that previous models had struggled to address, including the continuation of some bodily functions during sleep, the occurrence of dreams, and individual variations in sleep requirements.

### Physiological understanding of sleep

Ibn Sīnā's detailed analysis of the physiological effects of sleep demonstrated remarkable observational accuracy. He noted that “the predominant condition during sleep is that heat moves inward while cold appears externally, which is why sleepers need bedcovers for all their limbs more than those who are awake”.^28^^,^^29^ This observation of internal heat redistribution during sleep, while not entirely accurate in its mechanistic details, reflects a genuine attempt to understand sleep-related thermoregulatory changes—an area now confirmed by modern research^29^

Ibn Sīnā also described sleep's relationship with digestion, explaining that sleep strengthens natural bodily powers through the conservation of innate heat while relaxing psychic pathways through spirit moistening. He observed that sleep increases sweating compared to wakefulness when the body contains excess nutrition, indicating early recognition of the role of sleep in metabolic processes.^28^^,^^29^

### Sleep stages and cardiac monitoring

Perhaps most remarkably, Ibn Sīnā described three distinct sleep stages based on pulse changes, representing one of the earliest systematic attempts to characterize sleep phases through physiological monitoring. In the first stage, the pulse becomes small, weak, slow, and irregular as innate heat moves inward to assist digestion. During the second stage, the pulse becomes stronger and larger as the body gains energy from digested food, and heat begins redistributing outward. In the final stage, the pulse weakens again due to heat retention and waste accumulation.^30^

Ibn al-Nafīs's (1213–1288 CE) description of respiratory changes during sleep stages, as preserved in his *Sharḥ Tashrīḥ al-Qānūn* (Commentary on the Anatomy of the Canon), reveals a prescient attempt to correlate breathing patterns with sleep physiology. He observed that during initial sleep phases, breathing becomes “slower and less frequent but larger in volume,” attributing this to doubled respiratory control forces and the need for ventilation due to “internal gathering of innate heat”.^30^^,^^31^ In deeper sleep stages, he noted reduced air intake caused by “confinement of innate heat” and “accumulation of wastes”.^30^^,^^31^

Contemporary studies confirm that ventilation decreases during NREM sleep, particularly in slow-wave sleep (stage N3), with arterial carbon dioxide (CO_2_) levels rising by 2–3 mmHg due to reduced chemoreceptor sensitivity and diminished respiratory drive.^32^ This aligns partially with Ibn al-Nafīs's third stage, where reduced ventilation correlates with “waste accumulation,” a concept that can be seen as analogous to the mild retention of CO_2_ observed during sleep in modern physiology, although Ibn al-Nafīs framed this within humoral theory rather than respiratory gas exchange. His “innate heat” metaphorically parallels the metabolic activity that modern science links to oxidative processes influencing respiratory control.^33^

While Ibn al-Nafīs correctly identified sleep-stage-dependent breathing changes, his mechanistic explanations diverged from modern neurochemical models. The “doubled respiratory control forces” likely refers to medieval pneumatic theories of *rūḥ* (spirit) regulation rather than brainstem-mediated feedback loops. Similarly, “waste accumulation” encompasses broader humoral imbalances (e.g., phlegm, black bile) rather than specific gaseous metabolites like CO_2_. Nonetheless, his empirical focus on observable patterns—slower, deeper breaths transitioning to diminished intake—anticipates later discoveries about sleep-related hypoventilation (Fancy 2023c; West 2008).

Ibn al-Nafīs's work exemplifies the sophisticated empirical tradition of medieval Arab-Islamic medicine. By systematically linking pulse and respiratory changes to sleep stages, he advanced beyond Greco-Roman models, which primarily attributed sleep to brain cooling or humor redistribution.^2^^,^^30^ His emphasis on physiological monitoring (e.g., pulse, breathing depth) mirrors modern polysomnography's multiparameter approach, albeit without technological instrumentation.

### Humoral theory and sleep physiology

Medieval Arab-Islamic physicians developed sophisticated humoral frameworks linking the four classical elements, air, water, earth, and fire, to sleep patterns and disorders through systematic temperamental analyses. Ibn Sīnā categorized individuals into four temperaments with distinct sleep characteristics: hot and dry (choleric) temperaments experienced difficulty falling asleep and restless sleep, while cold and moist (phlegmatic) temperaments exhibited heavy sleep and difficulty awakening.^29^^,^^34^ Arab-Islamic physicians also recognized seasonal variations, recommending shorter sleep duration and cooler environments in summer (fire-dominant), and longer sleep periods with warmer conditions in winter (water/earth-dominant).^29^ Therapeutic interventions were tailored accordingly, including cooling foods and environments for hot temperaments and warming treatments for cold temperaments. This integrated humoral approach anticipated modern chronotype research and personalized sleep medicine by providing systematic frameworks for understanding individual sleep variations and developing targeted therapeutic interventions.

### Sleep hygiene and clinical recommendations

Ibn Sīnā’s *Canon of Medicine* (*al-Qānūn fī al-Ṭibb*) contains comprehensive sleep hygiene recommendations that remain remarkably relevant to contemporary practice. He emphasized that optimal sleep occurs after food has descended from the upper stomach and after the resolution of gas and belching, warning against sleeping before complete digestion due to its disruptive effects on both sleep quality and digestive processes.^28^, ^29^, ^30^

His recommendations included light walking before sleep if stomach emptying was delayed, avoiding sleep on either a completely empty or overly full stomach, and maintaining appropriate hydration levels.^35^ Ibn Sīnā noted that sleeping while thirsty was particularly problematic for individuals with hot, dry temperaments, while potentially beneficial for those with cold, moist constitutions.^30^^,^^36^

### Sleep position and respiratory function

Ibn Sīnā provided a detailed analysis of different sleep positions and their health effects, recommending initial sleep on the right side followed by transition to the left side. This recommendation, which aligns with prophetic traditions, was explained through an understanding of gastric anatomy and digestive physiology. Medical commentators explained that sleeping on the right side initially facilitates food descent to the stomach's fundus, which tilts rightward, while later transition to the left side provides optimal positioning for extended periods.^37^

Most significantly, Ibn Sīnā provided what appears to be the first accurate medical description of obstructive sleep apnea, noting that “those who sleep on their backs experience tongue relaxation backward, blocking the respiratory passage and causing snoring and choking”.^36^^,^^37^ This description precisely captures the pathophysiology of obstructive sleep apnea as understood in contemporary sleep medicine.

Ibn Sīnā also observed that back sleeping was common among sick and weak individuals due to muscle weakness preventing side positioning, and that such patients often slept with open mouths due to weakened jaw muscles. These observations provide classical descriptions of what modern medicine recognizes as obstructive sleep apnea risk factors and symptoms.

### Sleep disorders and mental health

Ibn Sīnā demonstrated sophisticated understanding of relationships between sleep and mental health, emphasizing that “healthy individuals must pay attention to sleep, ensuring it is moderate and appropriately timed, avoiding both excess and deficiency, and preventing prolonged wakefulness due to its negative effects on mental capabilities”.^37^ This holistic approach anticipated modern recognition of bidirectional relationships between sleep disorders and psychiatric conditions.^38^

Ibn Sīnā noted that excessive sleep leads to dulled psychic powers and brain heaviness, while sleep deprivation causes brain dryness, weakness of mental faculties, and humor burning leading to acute diseases.^37^ His observation that “excessive wakefulness corrupts brain temperament, inclining it toward dryness, weakens mental powers, and burns humors causing acute diseases” aligns closely with contemporary research on sleep deprivation's cognitive and physiological effects.^38^

### Pharmacological interventions

Ibn Sīnā’s *Canon of Medicine* includes a structured classification of neurological and sleep-related drugs, identifying agents that induce sleep, stupor, or calm mental agitation. He categorized them by their physiological and psychological effects, such as “anaesthetic,” “antirelaxant,” or “absorptive” drugs, demonstrating a proto-psychopharmacological model centuries before modern sleep medicine emerged.^27^^,^^39^^,^^40^

This classification represents one of the earliest systematic approaches to psychopharmacology, anticipating the development of modern sleep medications and their therapeutic applications.^27^^,^^39^ Ibn Sīnā’s recognition of different sleep medication categories reflects understanding that various sleep disturbances require different therapeutic approaches.

## Contributions of other scholars

### Before Ibn Sina (the 10th century)

**Ibn al-Jazzār (10th century CE),** a prominent physician from Kairouan in present-day Tunisia, authored *Kitāb al-Nawm wa-l-Sahar* (Book of Sleep and Sleeplessness), now lost.^41^ Nevertheless, his surviving writings in works such as “Provisions of the Traveler and Nourishment of the Resident” demonstrate a sophisticated understanding of the relationship between sleep and general health.

Ibn al-Jazzār provided detailed accounts of sleep-related disorders in his influential medical compendium *Zād al-musāfir wa-qūt al-ḥāḍir* (Provisions for the Traveler and Nourishment for the Sedentary). In his discussion of *subāt* (torpor), he described patients experiencing overwhelming sleepiness, sometimes even during the day, which he attributed to an excess of cold, phlegmatic humors *(an excess of the phlegm bodily fluid, according to humoral theory)* affecting the brain. While his framework was rooted in Galenic humoral theory, this clinical observation of excessive daytime sleepiness overlaps with what modern medicine recognizes as hypersomnia and, potentially, narcolepsy.^41^, ^42^, ^43^ Although Ibn al-Jazzār did not identify cataplexy as a distinct phenomenon, his descriptions of sudden weakness and loss of muscle control in the context of sleep disorders and epilepsy (*sarʿ*) are noteworthy. For example, he noted that epileptic patients might experience abrupt collapses and loss of bodily control, but he explicitly linked these episodes to seizures rather than emotional triggers, distinguishing them from cataplexy as currently defined.^41^^,^^43^

It is important to emphasize that medieval Arab-civilization physicians, including Ibn al-Jazzār, did not conceptualize narcolepsy or cataplexy as discrete clinical entities. Instead, symptoms that today might be classified under these diagnoses were categorized within broader syndromes such as *subāt* or *sarʿ* (*‘Sara’* in Arabic, meaning epilepsy), and explained according to prevailing humoral theories. The absence of a specific term or category for cataplexy highlights the differences in medical classification between the medieval and modern eras.^41^^,^^43^

In summary, while Ibn al-Jazzār's writings reveal an astute clinical awareness of sleep disorders and sudden motor disturbances, there is no direct evidence that he recognized narcolepsy with cataplexy as a distinct syndrome. His observations, however, demonstrate the sophistication of medieval Islamic medicine in describing and attempting to treat complex neurological and sleep-related conditions.^41^^,^^42^

**Ibn al-Ash'ath (died 975 CE)** recognized sleep as one of five fundamental factors influencing health and disease, alongside air quality, movement and rest, intake and elimination, and psychological states. This systematic framework demonstrates early recognition of sleep's integral role in maintaining physiological homeostasis.^44^

In the same treatise, Ibn Buṭlān (1001–1068 CE) his medical treatise *Taqwīm al-Ṣiḥḥah* explicitly warns that “excessive sleep causes brain obstruction and weakens memory,” echoing his broader concern for moderation in all physiological processes. He links overindulgence in sleep with cognitive dullness, reinforcing his belief that health preservation requires balance across all vital functions, including sleep.^45^

The contributions of al-Rāzī and Ibn Sīnā deserve particular attention due to their systematic approaches, detailed clinical observations, and lasting influence on subsequent medical practice both within the Islamic world and in medieval Europe through Latin translations.

### Post-Ibn Sīnā developments (11th–13th centuries)

#### Ibn al-Nafīs and active brain function during sleep

Ibn al-Nafīs (1213–1288 CE) made significant theoretical advances while maintaining Ibn Sīnā's pneumatic framework. He challenged earlier physicians who claimed that all brain activity ceases during sleep, demonstrating instead that only external sensation and voluntary movement stop, while internal senses continue functioning.^31^ Specifically, Ibn al-Nafīs explained that during sleep, external senses (such as sight and hearing) and voluntary movements are suspended, but internal faculties, like imagination and memory, remain active. He argued that the imaginative faculty (*al-quwwa al-mutakhayyila*) could even become stronger during sleep, as it is no longer distracted by external sensory input.^30^^,^^31^

Ibn al-Nafīs built on Ibn Sīnā’s metaphysical framework, which treats sleep as a state where the external faculties are suspended, allowing the internal faculties of imagination and memory to function autonomously,^46^ explaining that with the withdrawal of these senses, imagination is freed to operate more vividly, which explains the occurrence and intensity of dreams. He also noted that natural bodily functions, such as digestion and growth, are reinforced during sleep due to the body's inward focus on innate heat and energy.^30^^,^^31^

This nuanced framework is further elaborated in Ibn al-Nafīs's Sharḥ al-Mūjaz and related works, where he systematically addressed the continuity of internal mental faculties during sleep and discussed how dream phenomena arise from the unimpeded activity of the imaginative faculty. Modern historians recognize this nuanced view as an early precursor to the concept that sleep is a state of selective neural activation rather than global brain inactivity.^30^^,^^47^ Recent neuroimaging and EEG studies confirm that dreaming and memory processing are linked to persistent and sometimes heightened activity in specific brain regions during both rapid eye movement (REM) and non-rapid eye movement (10.13039/100016521NREM) sleep,^48^^,^^49^ supporting Ibn al-Nafīs's insight that the mind remains dynamically engaged during sleep.

##### Ibn al-Quff and sleep initiation mechanisms

Ibn al-Quff (died 1286 CE) provided innovative explanations for sleep initiation, building on and modifying Ibn Sīnā's pneumatic framework. He proposed that nature actively induces sleep by preventing the dissolution of internal fluids, which in turn relaxes the nerves and blocks the entry of spirit into the sensory and motor pathways.^50^ According to his theory, sleep results from an active, brain-initiated process that redirects the flow of the spirit, rather than from passive depletion of the spirit as earlier physicians had suggested. This model emphasized that the brain plays a central, dynamic role in initiating sleep, marking a significant departure from the more passive, humoral explanations of previous eras.^30^

Ibn al-Quff further argued that this process ensures the body's restorative functions are optimized during sleep, as the redirection of the spirit enables deeper physiological repair and mental rest. This conceptualization of sleep as an active, brain-mediated process represents a sophisticated understanding of sleep physiology, one that would not be fully appreciated in Western medicine until the modern era.^30^

This conceptualization of sleep as an active brain-mediated process represents a sophisticated understanding that would not be fully appreciated in Western medicine until the modern era.

## Institutional networks and knowledge transmission in Medieval Arab-Islamic Sleep Medicine

A comprehensive examination of Ibn Abī Uṣaybiʿah's *ʿUyūn al-anbāʾ fī ṭabaqāt al-aṭibbāʾ* in the mid-13th century supports the view that prominent medieval Arab-Islamic physicians such as al-Rāzī and Ibn Sīnā operated within intellectually vibrant and institutionalized environments. Al-Rāzī served as chief physician in Baghdad and Rayy, where he was “called upon by Caliph Al Muktafi to be the chief director of the largest hospital in Baghdad” and “appointed as director of the hospital of his hometown Al Rayy during the reign of Mansur Ibn Ishaq Ibn Ahmad Ibn Asad of the Samanian dynasty”.^51^ Ibn Sīnā practiced in Bukhara and moved between various eastern Islamic locations, including Gurganj, as documented in his autobiography: “Then al-Natili left me, going on to Gurganj, opposite Khwarazm, seeking the court of the Khwarazm-shah Ma'mun ibn Muhammad” (Gohlman, 1974). These physicians wrote their contribution in Arabic and were often associated with major hospitals (bīmāristāns), particularly the Nāṣirī hospital in Cairo and the al-Nūrī hospital in Damascus, which served not only as treatment centers but also as academic hubs for collaborative research, clinical observation, and theoretical advancement in medical sciences, including sleep-related disorders.^52^

The educational model evident in *Uyūn al-anbāʾ* reflects a form of hospital-based clinical apprenticeship, where students observed seasoned physicians treating a range of conditions, including those related to sleep. This structured pedagogy, anchored in practical observation and oral instruction, facilitated the systematic transmission of medical knowledge across generations.^52^ The presence of such training mechanisms helps explain the conceptual consistency and sophistication of medical knowledge in the medieval Islamic world, which emerged not from isolated genius but from an enduring institutional framework.^53^

Additionally, the biographies of physicians in *Uyūn al-anbāʾ* frequently portray them as polymaths integrating astronomy, psychology, and theology into their medical practice. Figures such as Ibn Riḍwān, for instance, employed astrological charts and planetary models to assess health and circadian rhythms, highlighting the holistic approach characteristic of Islamic medicine. Sleep was viewed not as a purely physiological state but as a condition influenced by cosmic, spiritual, and environmental forces, embedded within a wider framework of human well-being.^52^

The institutional framework of medieval Islamic medicine created conditions that allowed scholars from diverse ethnic backgrounds to contribute to a shared intellectual tradition. Hospital-based medical education, standardized Arabic-language curricula, and systematic knowledge exchange networks enabled the development of sophisticated medical understanding that transcended individual ethnic or regional limitations. This institutional integration explains how innovations by Persian-origin scholars, such as Ibn Sīnā, could immediately influence Arabic-speaking colleagues in Baghdad, Damascus, and Cairo, thereby creating a unified tradition of medical practice and research.^54^

## Dreams in Arab-Islamic medical and philosophical tradition

Medieval Arab-Islamic approaches to dreams integrated empirical observation with spiritual understanding, creating comprehensive systems for dream analysis that influenced both medical practice and popular culture for centuries. Dreams and visions were considered important not only in religious life but also in historiography and medicine, reflecting a unique cultural embeddedness of the imagination.

### Theological foundations

Islamic dream theory derives from Quranic and prophetic teachings that recognize different categories of dreams. The Prophet Muhammad said: “A good dream is from Allah, and a bad dream is from Satan. So whoever has seen (in a dream) something he dislikes, then he should spit without saliva, thrice on his left and seek refuge with Allah from Satan, for it will not harm him, and Satan cannot appear in my shape.”.^55^ Additionally: “The (good) dreams of a faithful believer is a part of the forty-six parts of prophetism”.^56^

This theological framework encouraged systematic dream analysis while maintaining clear distinctions between spiritually significant dreams and ordinary psychological phenomena. The Qur'an itself upholds the validity of dream experiences, recounting the dreams of prophets such as Yusuf (Joseph) and Ibrahim (Abraham), and emphasizing their role in revelation and guidance.^3^

### Ibn Sīnā's dream psychology

Ibn Sīnā’s dream theory integrated his pneumatic sleep model with a sophisticated psychological analysis. In his philosophical works, particularly *al-Shifā’* (The Metaphysics of the Healing), he explained that the imaginative faculty (*al-quwwa al-mutakhayyila*) continues operating during sleep, using stored sensory images and previously perceived meanings to create new dream imagery.^46^^,^^57^ Ibn Sīnā proposed that during sleep, when the soul is less occupied with external sensory input, it becomes more receptive to meanings from the “Active Intellect” (*al-‘aql al-fa‘āl*) or unseen realm, translating these into dream symbols through the imaginative faculty.^57^ This framework enabled him to explain both ordinary dreams reflecting daily experiences and seemingly prophetic dreams containing information not previously known to the dreamer.^57^^,^^58^

While Ibn Sīnā did not explicitly use the modern term “lucid dreaming,” he did note that individuals with strong and well-trained imaginative faculties could, during sleep, recognize the dream state and exercise some degree of control over dream content.^59^ Recent scholarship interprets these passages as an early description of lucid-like dreaming, where the dreamer is aware of dreaming and can direct dream imagery according to intellectual or knowledge-seeking needs.^57^ Modern neuroscience has confirmed that lucid dreaming is a distinct state involving metacognitive awareness and voluntary control during REM sleep, and recent studies continue to explore its cognitive and therapeutic implications.^60^^,^^61^ Recent scientific reviews confirm that lucid dreaming is a distinct state involving metacognitive awareness and voluntary control during REM sleep, and highlight the long-standing interest in lucid and conscious states during sleep in both Islamic and other religious traditions.^62^

### Systematic dream classification

Arab-Islamic physicians developed comprehensive dream classification systems that demonstrated sophisticated psychological insight. Al-Rāzī and Ibn Sīnā, building upon earlier Islamic scholarly traditions, classified dreams into three categories: true visions (*ru'ya*) with spiritual significance, psychological dreams (*hulm*) resulting from internal preoccupations, and somatic dreams arising from physical temperament imbalances (*dyscrasia*).^63^, ^64^, ^65^, ^66^, ^67^

This tripartite Islamic classification anticipates modern frameworks in dream psychology: ‘true’ dreams align with cognitive/problem-solving types, psychological dreams mirror affective or emotional content, and physical dreams resonate with somatic and health-related interpretations.^68^, ^69^, ^70^

### Dreams as diagnostic tools

Arab-Islamic physicians innovatively used dream content for medical diagnosis, integrating dream analysis with humoral theory. Dreams of fire or flying indicated excessive yellow bile; dark, depressing dreams suggested black bile dominance; dreams of water and cold reflected phlegm excess; while bloody or red imagery indicated blood predominance.^71^

This systematic approach to dream-based diagnosis represents early recognition of psychosomatic relationships, anticipating modern psychosomatic medicine's understanding of mind-body connections.^72^

### Ibn Khaldun's scientific approach

Ibn Khaldun (1332–1406 CE) addressed dream interpretation as a *distinct science* in his seminal work, *The Muqaddimah*.^73^ There, he systematically classified dreams into three categories: *true dreams from God* requiring no interpretation, *symbolic dreams from angels* requiring interpretation, and *confused dreams from Satan* that are considered meaningless. This tripartite model reflects a structured epistemology that combines metaphysical and rational insights.

His integration of dream theory into broader discussions on prophecy, imagination, and intellectual faculties suggests a proto-scientific methodology, anticipating later analytical approaches. Although rooted in Islamic theology, his classification and analysis reflect a systematic reasoning process analogous to modern cognitive frameworks. As Shaikh and Pruett argue,^74^^,^^75^ Ibn Khaldun's work exemplifies a rational historiographical project that accommodates divine knowledge, spiritual intuition, and natural observation within a unified schema.

This scientific approach to dream categorization demonstrates systematic methodology that influenced subsequent Islamic scholarship and parallels modern psychological approaches to dream analysis.

## Clinical and therapeutic applications

### Integration of sleep medicine with general healthcare

Arab-Islamic civilization physicians pioneered integrated approaches to sleep medicine that considered sleep within comprehensive healthcare frameworks.^19^^,^^37^ Rather than treating sleep disorders in isolation, they systematically addressed relationships between sleep disturbances and systemic diseases, dietary factors, environmental conditions, and psychological states.^1^^,^^13^

This holistic approach anticipated modern sleep medicine's recognition that effective treatment often requires addressing multiple contributing factors rather than focusing solely on sleep symptoms.^23^

### Preventive sleep medicine

Arab-Islamic medical texts emphasized preventive approaches to sleep disorders through lifestyle modification, environmental optimization, and behavioral interventions.^14^^,^^29^ These recommendations included sleep schedule regulation, appropriate nutrition timing, physical activity guidelines, and stress management techniques.

Such comprehensive preventive approaches align closely with contemporary sleep hygiene recommendations and cognitive-behavioral therapy for insomnia,^38^ demonstrating remarkable continuity between historical and modern therapeutic strategies.^25^^,^^26^

### Pharmacological innovations

While limited by available materials, Arab-Islamic civilization physicians developed a sophisticated understanding of sleep-affecting substances, creating detailed classifications of herbal and mineral preparations for sleep disorders.^19^^,^^37^ Al-Majusi's systematic division of drugs into categories, including hypnotics and sedatives, exemplified their methodical approach to pharmacological classification. Their systematic approach to medication categorization, dosage guidelines, and pharmaceutical techniques established foundations for modern psychopharmacology.^27^^,^^39^

## Legacy and modern relevance

### Influence on European medicine

The extensive translation of Arab-Islamic medical texts into Latin during the 12th and 13th centuries brought sophisticated sleep medicine concepts to European universities. Ibn Sīnā's “Canon” remained a standard textbook in European medical education for over four centuries, ensuring that Arab-Islamic sleep theories influenced Western medical development well into the early modern period.^76^^,^^77^

This transmission represents one of the most significant examples of cross-cultural scientific exchange in medical history, demonstrating how Islamic scholarly traditions shaped the foundations of modern European medicine.

### Contemporary validation

Many observations and recommendations made by medieval Arab-Islamic civilization physicians have been validated by contemporary sleep research. Their recognition of sleep's immune function, understanding of the importance of circadian rhythm, appreciation of dietary influences on sleep quality, and emphasis on sleep environment optimization align with current evidence-based sleep medicine practices.^78^, ^79^, ^80^

Table 3 systematically documents the major breakthroughs in sleep science by medieval Arab-Islamic civilization scholars, revealing how these contributions established foundational principles across multiple domains of sleep medicine that continue to influence contemporary practice.

**Table 3:** Major Breakthroughs in Sleep Science by Medieval Arab-Islamic Civilization Scholars.

Scholar	Time Period	Major Breakthrough/Contribution	Modern Relevance
**Al-Rāzī (Rhazes)**	865-925 CE	Clinical use of sleep patterns as diagnostic tools	Anticipates modern understanding of sleep's role in immune function, healing processes, and recovery monitoring
**Al-Rāzī**	865-925 CE	Experimental methodology in sleep research	Early controlled clinical trial methodology with comparison groups, predating Western adoption by centuries
**Al-Rāzī**	865-925 CE	First detailed clinical description of sleep paralysis	First comprehensive medical documentation of sleep paralysis symptoms and dual classification system
**Ibn al-Jazzār**	10th century CE	Clinical description of hypersomnia (subāt)	Provided early clinical observations of excessive daytime sleepiness, corresponding to modern hypersomnia disorders
**Ibn Sīnā (Avicenna)**	980-1037 CE	Pneumatic theory of sleep	First comprehensive theoretical framework explaining sleep-wake mechanisms through psychic spirit (ruh nafsani)
**Ibn Sīnā**	980-1037 CE	Three-stage sleep classification	Earliest systematic attempt to characterize sleep phases through physiological monitoring using pulse changes
**Ibn Sīnā**	980-1037 CE	First accurate description of obstructive sleep apnea	Precisely captures modern understanding of upper airway obstruction pathophysiology and positional therapy
**Ibn al-Nafīs**	1213-1288 CE	Active brain function during sleep theory	Anticipated modern understanding of selective neural activation and continued cognitive processing during sleep
**Ibn al-Nafīs**	1213-1288 CE	Respiratory changes during sleep stages	Sleep-stage-dependent breathing patterns with CO_2_ retention during NREM sleep confirmed by modern polysomnography
**Ibn al-Quff**	died 1286 CE	Active sleep initiation theory	Anticipated modern understanding of sleep as an active, brain-mediated neurobiological process rather than passive state
**Various scholars**	9th-13th centuries	Tripartite dream classification	Systematic categorization anticipating modern psychological frameworks for dream analysis and REM sleep research

This concordance between historical Islamic medicine and modern research suggests that careful empirical observation, even without advanced technology, can yield lasting insights into human physiology and therapeutic interventions.

### Methodological contributions

Perhaps most significantly, Arab-Islamic civilization physicians established methodological approaches to sleep research that remain relevant today.^81^^,^^82^ Their emphasis on systematic clinical observation, experimental comparison groups, integration of multiple data sources, and comprehensive therapeutic approaches established principles that continue to guide contemporary sleep medicine research and practice.

## Research limitations

This review of medieval Arab-Islamic civilization contributions to sleep medicine acknowledges several important methodological constraints that readers should consider when interpreting our findings. Many historically significant texts remain unpublished, existing only in private collections or institutional archives with restricted access. Our analysis necessarily focuses on available critical editions and published manuscripts, which may not provide a comprehensive historical picture. The catastrophic destruction of Baghdad's libraries in 1258 CE during the Mongol invasion resulted in the loss of irreplaceable manuscripts, meaning our review likely underrepresents the actual breadth of medieval Islamic contributions to sleep medicine.

Translation challenges arise from the complex medieval Arabic medical terminology that often lacks direct modern equivalents, requiring interpretive translation that may introduce subtle variations in meaning. Technical terms like “rūḥ nafsānī" (psychic spirit) represent complex concepts that resist precise modern translation. The 700- to 1000-year gap between the original composition and contemporary analysis creates inevitable interpretive challenges, particularly regarding the practical application of historical therapeutic recommendations. Our focus on well-preserved, frequently cited texts may overrepresent prominent scholars while potentially underrepresenting innovative work by less famous practitioners whose writings have been lost.

## Conclusion

This analysis demonstrates that the scientific achievements documented herein emerged from a unified civilizational framework that successfully integrated diverse ethnic, cultural, and intellectual traditions under the successive Arab Islamic caliphates. Beginning with the Umayyad Caliphate (661–750 CE) and flourishing under the Abbasid Caliphate (750–1258 CE), this civilizational matrix established the institutional and linguistic foundations that enabled remarkable medical advances. The Arabic language provided the medium for scholarly discourse across the empire, Islamic institutions created the educational infrastructure centered around major hospitals and libraries, and collaborative networks enabled knowledge exchange across vast geographical regions extending from Al-Andalus to Central Asia. The Abbasid period, in particular, witnessed the establishment of the House of Wisdom (Bayt al-Hikma) in Baghdad and the systematic translation movement that preserved and expanded upon Greek, Persian, and Indian medical knowledge. This civilizational approach to historical analysis, standard in studies of other major intellectual traditions, provides the most accurate framework for understanding how diverse scholars contributed to the remarkable advances in sleep medicine during this period.

Within this unified framework, the comprehensive analysis demonstrates that medieval Arab-Islamic civilization scholars made foundational contributions to sleep science that have been inadequately recognized in conventional historical accounts. The systematic contributions of medieval Arab-Islamic physicians to sleep science can be categorized across multiple domains, as outlined throughout this review. These comprehensive overviews demonstrate the remarkable sophistication and clinical accuracy of their observations, many of which anticipated modern sleep medicine concepts by several centuries. Rather than merely preserving ancient Greek knowledge, these physicians developed original theoretical frameworks, conducted systematic clinical observations, and established therapeutic approaches that anticipated many concepts in modern sleep medicine.

The pneumatic theory of sleep developed by Ibn Sīnā provided a mechanistic explanation for sleep-wake transitions that remained influential for centuries. Arab-Islamic physicians' clinical descriptions of sleep disorders, including sleep paralysis, hypersomnolence, description of the symptoms of sleep apnea, and insomnia, demonstrated remarkable observational accuracy. Their systematic approaches to sleep hygiene, dream interpretation, and integrated medical care established principles that continue to inform contemporary practice. The historical record reveals that this Arab-Islamic civilizational contribution to sleep science occurred during a period often overlooked in Eurocentric academic narratives. These contributions represent more than historical curiosities—they demonstrate the value of diverse intellectual traditions in advancing scientific knowledge and the importance of cross-cultural exchange in medical development.

Recognition of these historical contributions serves multiple purposes: it provides a more complete and accurate account of sleep science history, demonstrates the continuity between traditional and modern medical approaches, and highlights the value of integrating diverse cultural perspectives in contemporary medical education and research. As modern sleep medicine continues evolving, the medieval Arab-Islamic tradition offers valuable lessons about holistic approaches to sleep health, the importance of careful clinical observation, and the benefits of integrating multiple therapeutic modalities. These historical insights remain relevant for contemporary efforts to address the growing burden of sleep disorders in modern societies.

Future research should prioritize rigorous scientific validation of Arab-Islamic civilization contributions to sleep science through systematic examination of original manuscripts and primary sources. Rather than relying solely on secondary interpretations, scholars must engage directly with medieval Arabic texts to authenticate historical claims and uncover previously overlooked insights. This approach requires collaboration between historians, Arabic language specialists, and sleep medicine experts to ensure accurate translation and contextual understanding of these foundational works.

This historical analysis ultimately demonstrates that the development of sleep medicine represents a truly global endeavor, with significant contributions from diverse civilizations and cultural traditions. Recognizing this intellectual diversity not only corrects historical omissions but also enriches our understanding of sleep science and its potential for future development.

## Ethical approval

Not applicable. This was an analysis of the literature and guidelines development.

## Consent

Not applicable.

## Authors’ contribution

Single author.

## Source of funding

This work was supported by the Strategic Technologies Program of the National Plan for Sciences and Technology and Innovation in the KSA, Riyadh, KSA.

## Declaration of competing interest

The author has no conflict of interest to declare.
